# Radial Head Fracture (Mason Type 4) Fixation With Headless Compression Screws: The “Tripod Technique”

**DOI:** 10.7759/cureus.67576

**Published:** 2024-08-23

**Authors:** Jitesh P Phadatare, Sushil Mankar, Vismay V Harkare, Rahul H Sakhare, Harsh B Thakkar

**Affiliations:** 1 Orthopaedics, N. K. P. Salve Institute of Medical Sciences and Research Centre, Nagpur, IND; 2 Orthopaedics and Traumatology, N. K. P. Salve Institute of Medical Sciences and Research Centre, Nagpur, IND; 3 Orthopaedics and Traumatology, N. K. P. Salve Institute of Medical Sciences and Research Centre and Lata Mangeshkar Hospital, Nagpur, IND

**Keywords:** mason classification, unstable elbow fracture dislocation, tripod technique, headless compression screws, radial head fracture

## Abstract

Radial head fractures are fairly common fractures in the general population accounting for up to 30% of elbow fractures. The management of these fractures is controversial, specially in the higher grade of fractures. The current case report presents a middle-aged male patient with a fracture dislocation of the radial head in the dominant hand. After undergoing adequate investigations, the fracture was classified and managed with headless compression screws using the tripod technique. The management of the patient, preoperative planning, and the complications faced are mentioned in the current report. The patient on follow-up shows a good range of motion and an improved Mayo elbow score. Thus, stating good results can be obtained in Mason type 4 fractures using osteosynthesis with headless compression screws.

## Introduction

Fractures of the radial neck and head amount to 2%-5% and 30% of elbow fractures, respectively [1]. Radial head fracture is seen in 25 of 100,000 adults. It is more common in younger patients [2]. The fractures have more predilection toward females, and the most common mode of trauma is falling on an outstretched and pronated hand [3]. One of the major secondary constraint of the elbow joint is the head of the radius and especially in an elbow which is deficient of a medial collateral ligament (MCL) [2].

It was Mason who originally classified radial head fractures, and his classification is the most frequently used classification for radial head fractures. They classified the fractures according to the severity of the involvement with type I fractures being undisplaced fractures, type II fractures being displaced with more than 2 mm)of articular involvement, type III being comminuted fractures, and type IV included fractures associated with dislocation [3].

Various treatment modalities have been used over the past to address the fracture. The use of plates for internal fixation following open reduction, arthroplasty of the radial head, and fixation with headless compression screws are usually employed methods for the management of Mason type 4 fractures. Depending on the presentation of the fracture, radial head excision can be considered as a treatment modality depending on the demands of the patients but is associated with its own set of complications [4].

Radial head fractures should be meticulously managed to avoid unfavorable outcomes such as stiffness, post-traumatic arthritis, and joint instability [4]. Different presentations of the radial neck and head anatomy among different individuals result in the inefficient fitting of the plates, which results in inadequate reconstruction, poor reduction, and stiffness of the elbow joint [2].

However, when there is severe comminution of the radial head, excision of the radial head without arthroplasty can be done, but it is usually associated with increased incidence of valgus instability and elbow joint stiffness following the excision of radial head [3,5]. In cases of unreconstructable, comminuted fractures of the head of radius, arthroplasty is suitable [2]. 

Headless compression screw was first made in Australia in 1984. It was used for the scaphoid fracture fixation at the start. Its use was increased because of properties like decreased periosteal stripping and impingement as it lacks the screw head and gets submerged under the cartilage. The versatility of its use gradually increased over the time [2].

The tripod technique comprises the use of three orthogonally directed compression screws which are headless for fixation of the fragments and reduce the head part on the neck part. The passage of one screw in a longitudinal manner from the head region into the distal region provides the axial stability to the fixation. As a result, the construct looks like a tripod and gives stability to fractures which are unstable in both axial plane and coronal plane. Buried screws provide a superiority to the construct which is biologically as well as mechanically superior and prevents impedance of the proximal radioulnar joint motion [4].

The current case report mentions about the management of a Mason type 4 fracture of the radial head in the dominant (left) hand of a middle-aged male. The planning done and the challenges faced are mentioned in the report.

## Case presentation

Patient information

A 43-year-old middle-aged man had come to us with a traumatic history due to a fall. The patient had come with intense swelling over the elbow region with an inability to move the elbow joint of the dominant (left) hand.

Clinical findings

The patient was examined thoroughly. He had pain and swelling over the left elbow joint. He had a painful and restricted range of motion around the elbow joint with obvious deformity seen. The three-point bony relation was found to be disturbed. There was no distal neurovascular deficit. The olecranon process was markedly prominent posteriorly, and hence, a clinical diagnosis of elbow dislocation was made.

Timeline

A 43-year-old male patient who was left-hand dominant had come with a restricted elbow range of motion following trauma that occurred three days back. The patient first went to a quack nearby where he was managed with medications. The patient came to our institute three days later with persistent pain and deformity. The patient had undergone investigations like an X-ray in the casualty itself which revealed a fracture of the radial head on the left side with elbow dislocation. The patient’s elbow was reduced with traction, countertraction, and manipulation.

Postreduction X-ray was done, and an above elbow slab was given. The patient was then scheduled for surgery, which was internal fixation following open reduction with the use of three headless compression screws in a tripod fashion. Postoperatively, subject had an improved range of motion with up to 110° flexion at the elbow joint. A marked improvement in the forearm supination and pronation was noted. At the one-year follow-up, the patient had a good functional range of motion and was able to get back to his livelihood.

Diagnostic assessment

**Figure 1:**
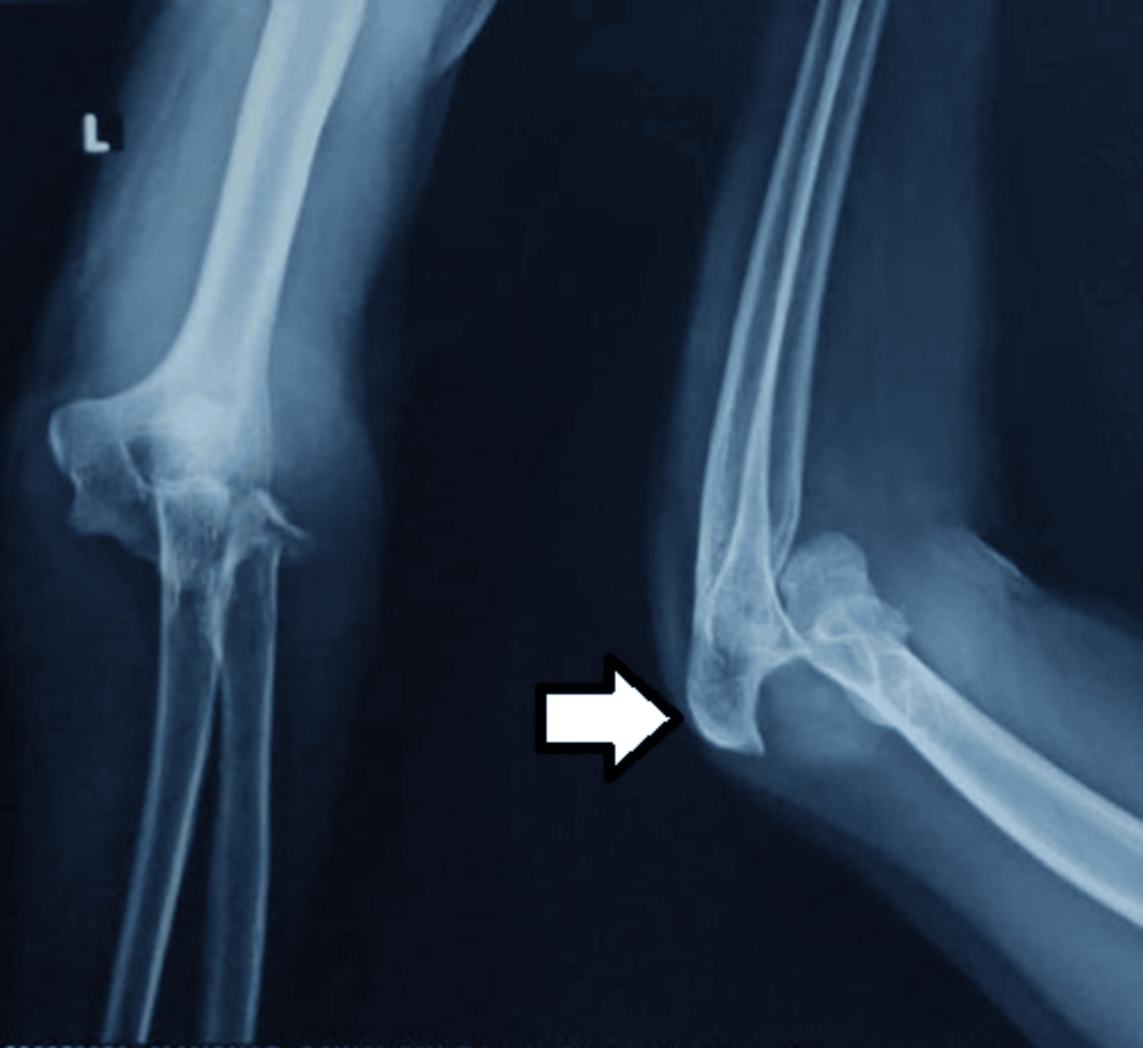
Prereduction AP and lateral radiographs showing elbow dislocation (white arrow) AP: Anteroposterior

The patient was advised a plain radiograph of the left elbow joint which confirmed the clinical diagnosis of elbow dislocation, which was posterior with the fracture of the head of the radius and its neck (Figure 1).

**Figure 2:**
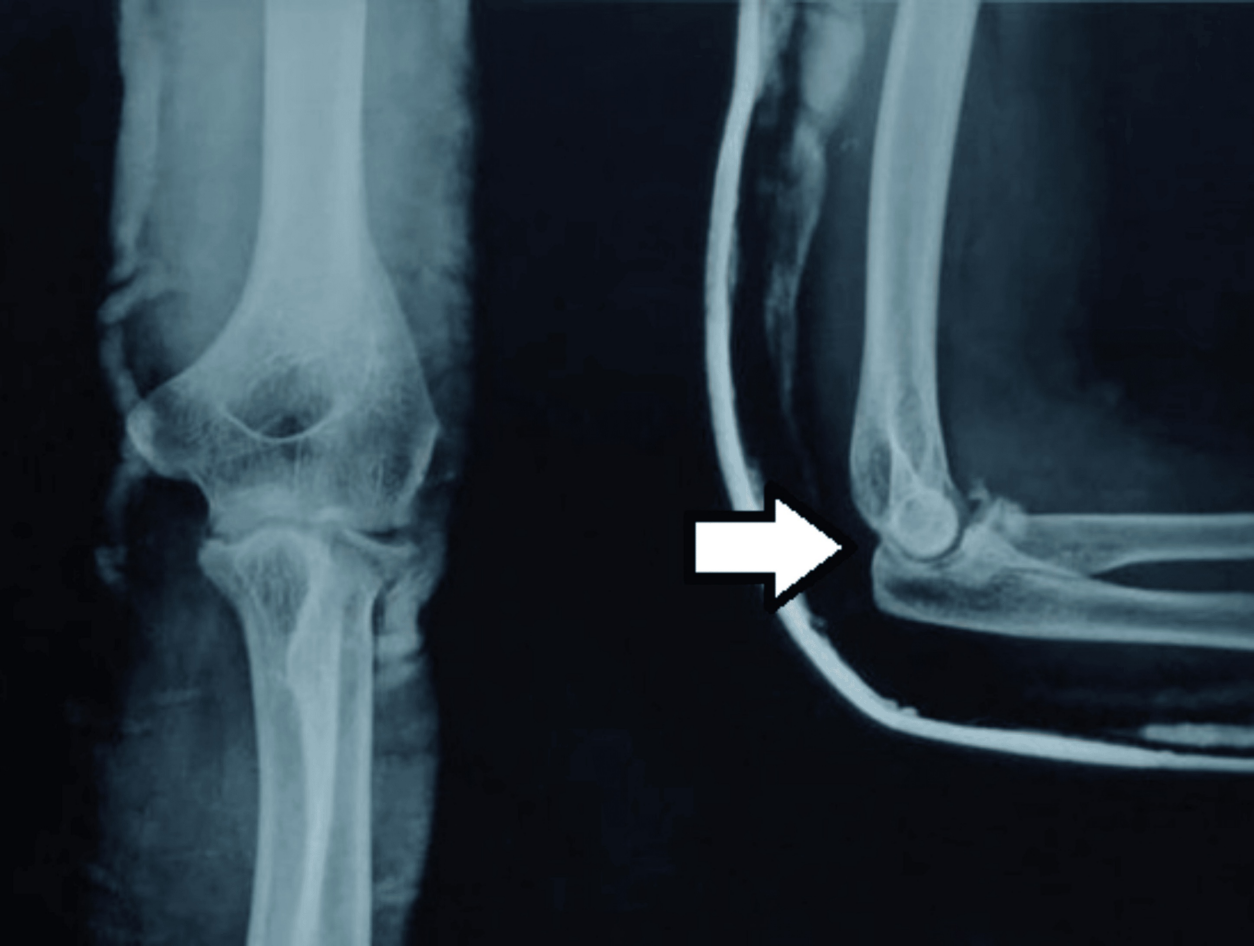
Postreduction AP and lateral radiographs of left elbow joint depicting reduction of elbow dislocation (white arrow) AP: Anteroposterior

The elbow was relocated using a closed reduction maneuver, and a postreduction X-ray was done to rule out the terrible triad of the elbow joint. No evidence of the coronoid process fracture was seen on the postreduction X-ray (Figure 2). After reduction, tests to check for elbow instabilty were performed and found to be within normal limits.

Intervention

**Figure 3:**
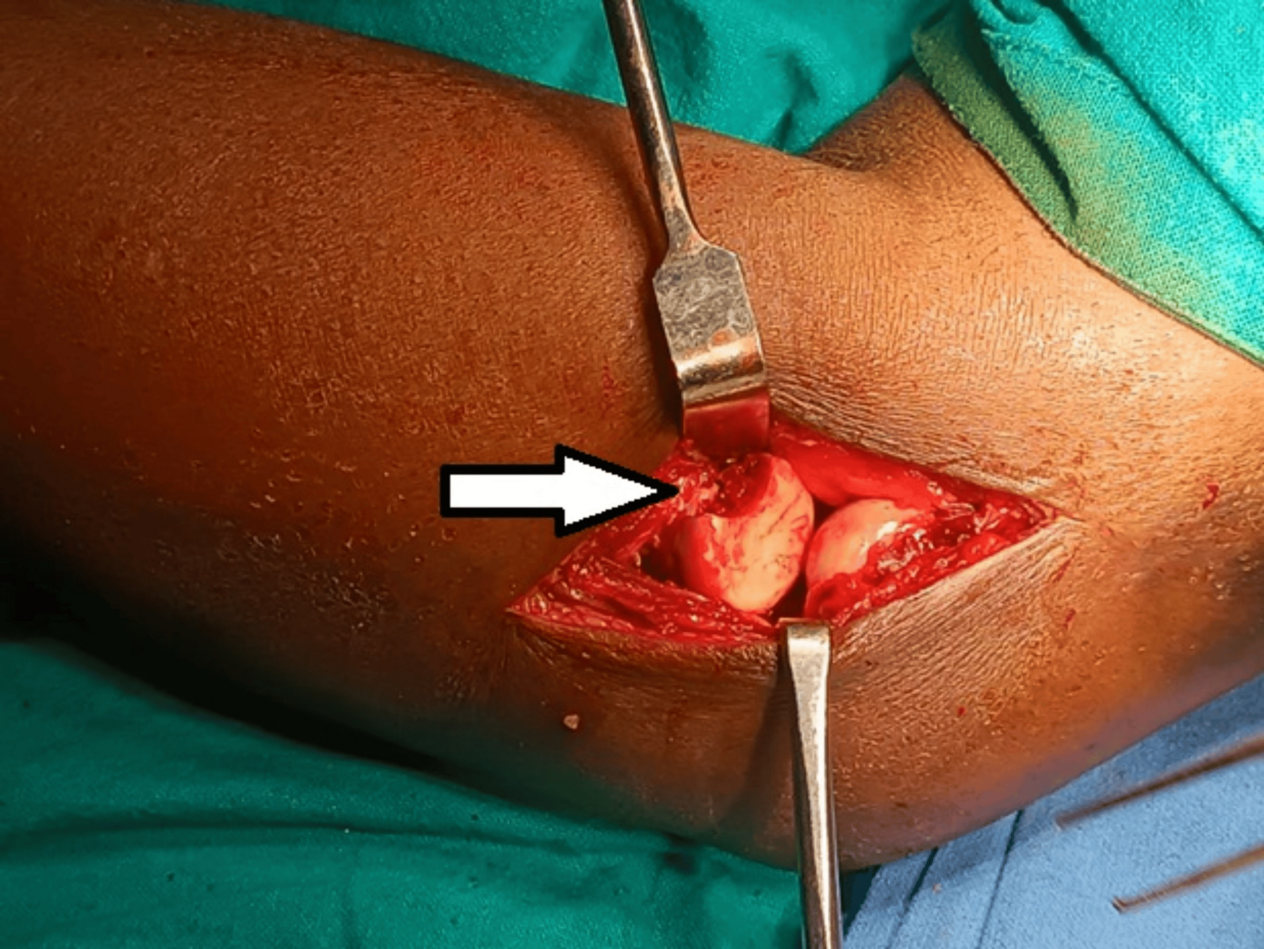
Intraoperative image showing a radial head fracture

**Figure 4:**
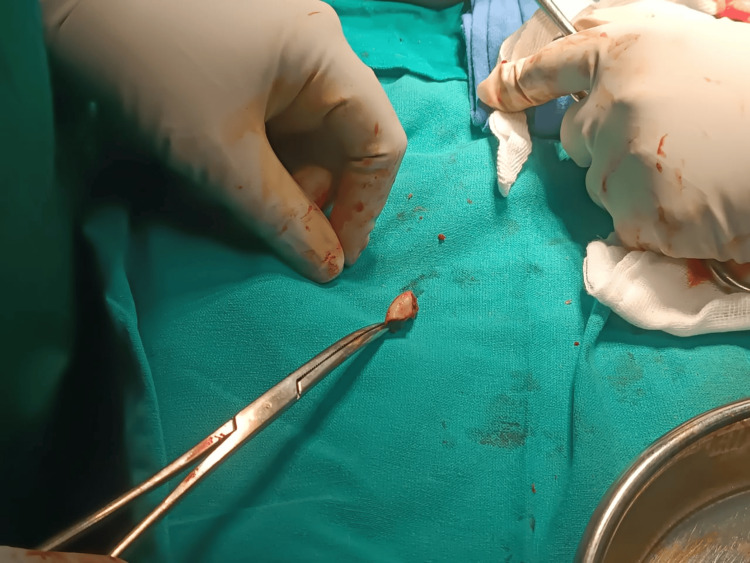
Intraoperative image showing fracture fragment of the radial head

**Figure 5:**
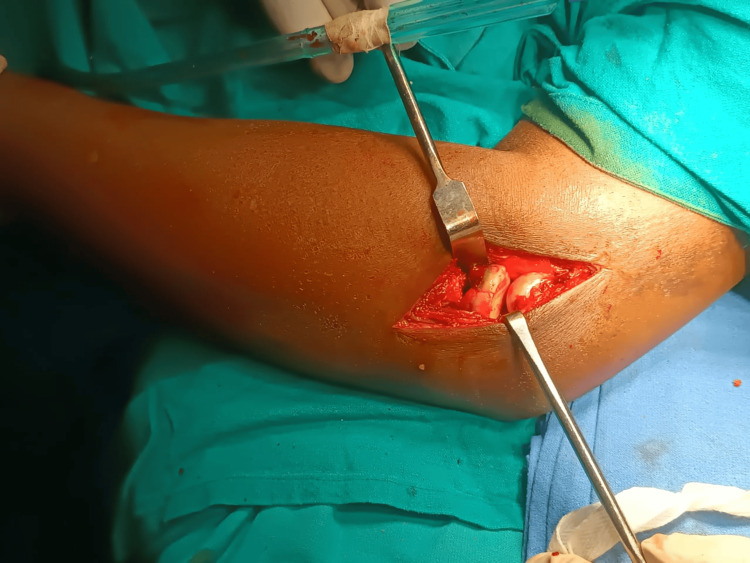
Intraoperative image showing reduction of the radial head fracture fragment

**Figure 6:**
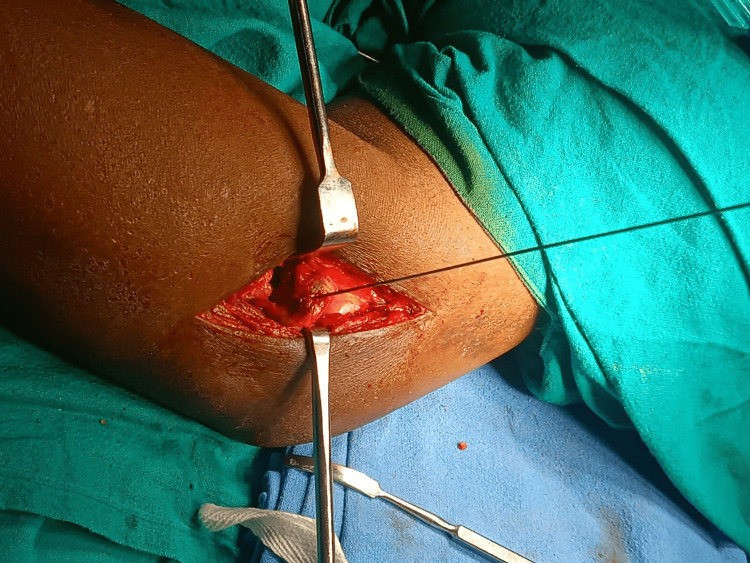
: Intraoperative image showing the passage of the guidepin through reduced radial head fracture fragments

**Figure 7:**
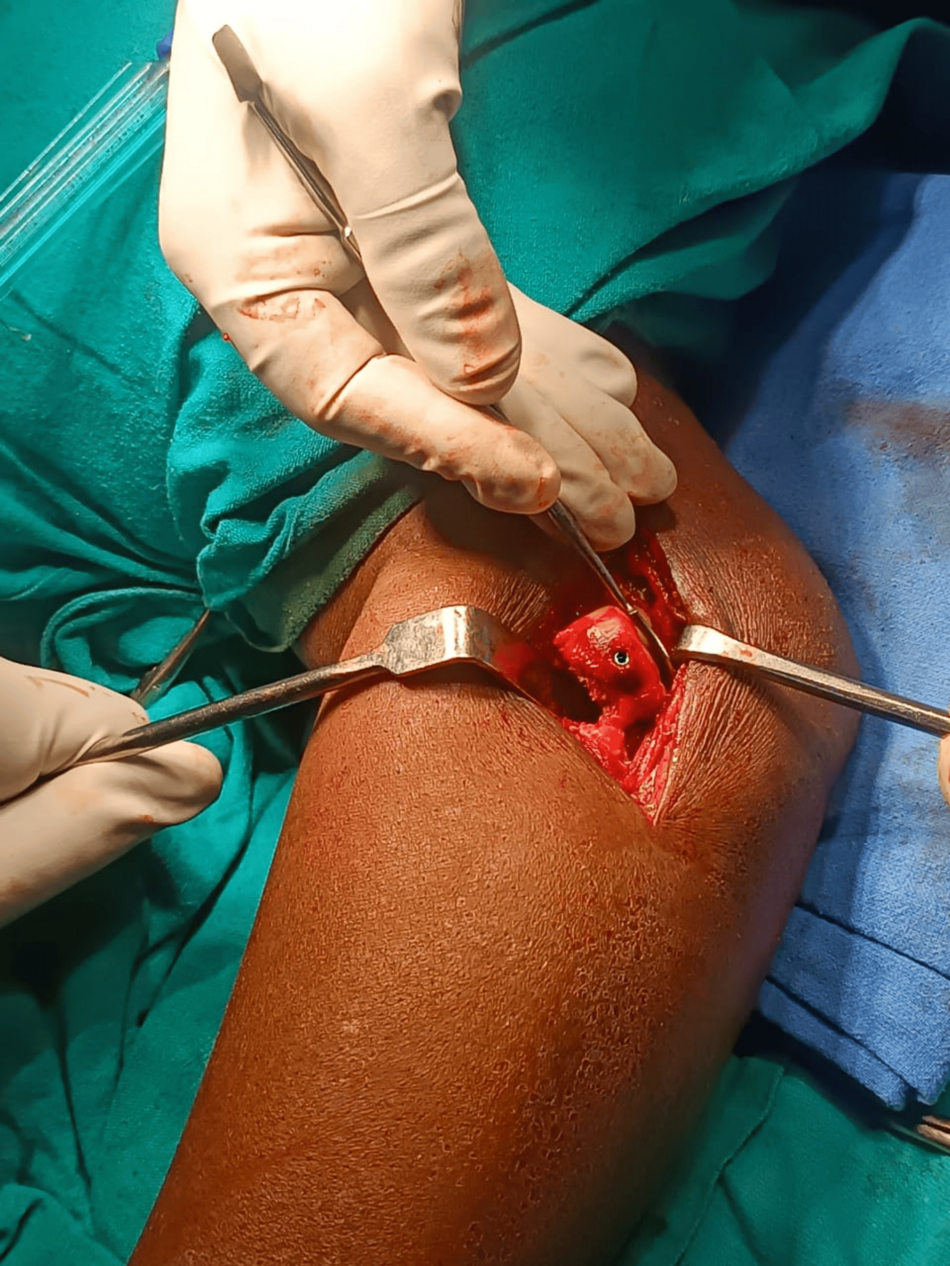
Intraoperative image showing reduction of fracture of the radial head with headless compression screw

**Figure 8:**
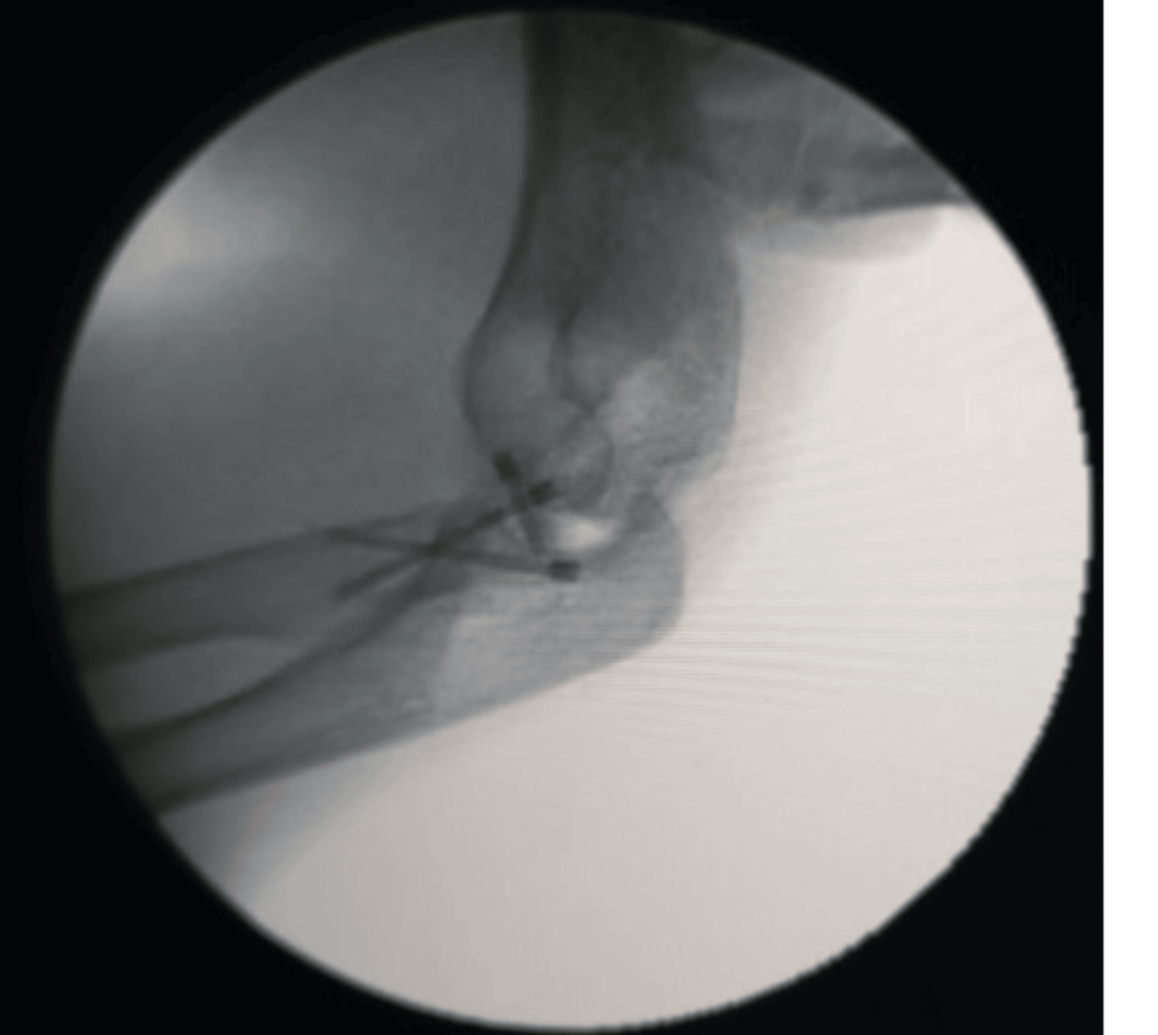
C-arm image showing lateral view of the final reduction of the radial head fracture with the help of three headless compression screws

**Figure 9:**
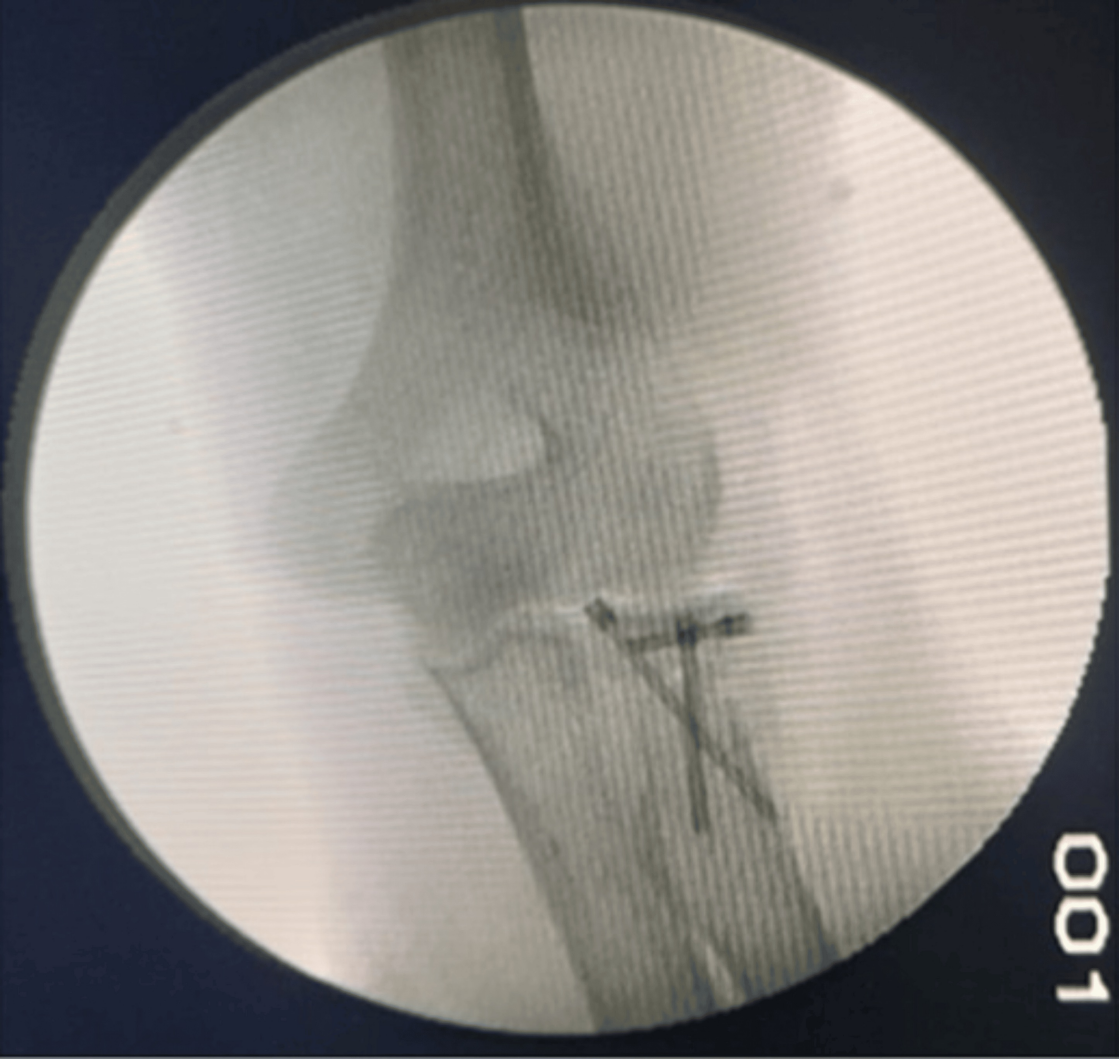
C-arm image showing AP view of the final reduction of the radial head fracture using three headless compression screws AP: Anteroposterior

**Figure 10:**
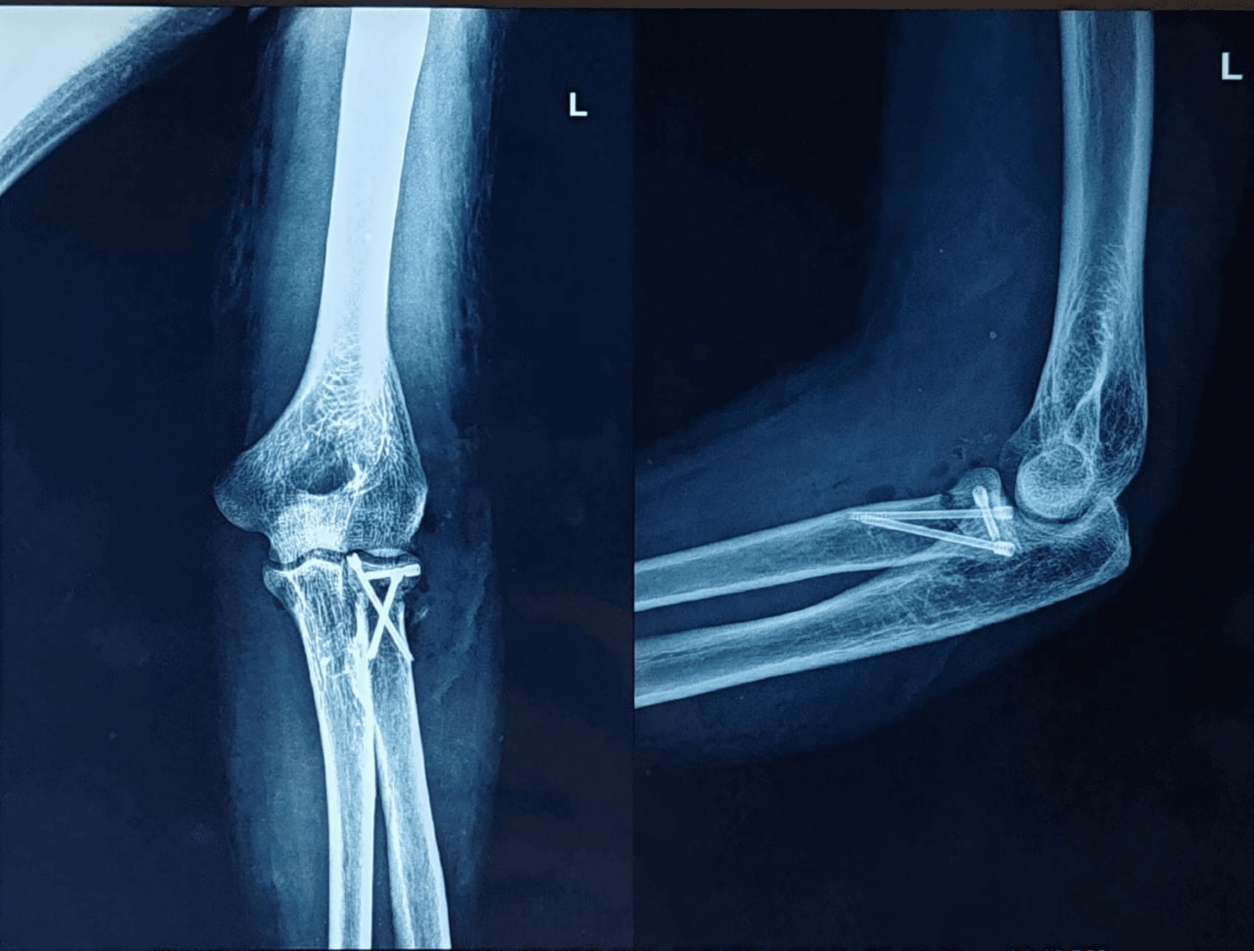
Immediate postop X-ray showing AP and lateral views of the left elbow joint with the radial head fracture reduced with the help of three headless compression screws AP: Anteroposterior

Upon studying the X-rays, a diagnosis of type 4 Mason fracture was established. The patient was planned for internal fixation after open reduction of the head of the radius in a tripod manner with headless compression screws. A backup plan to fix the fracture using a plate following open reduction of the fracture or excision of the radial head was also kept. Radial head prosthesis could not be kept as a backup plan due to the financial constraints of the patient. The patient was placed supine on the operating table (OT), and the standard lateral approach with the Kocher’s interval was planned for the patient. On performing the exposure, the radial head was found to be fractured in three parts (Figure 3). Reduction of the fragment was tried in situ but was not adequately achievable; hence, the radial head fragments were extracted on the OT, and reduction was attempted (Figure 4). The reduction was achieved by passing a transverse headless compression screw of size of 2 x 20 mm in the two fragments of the radial head (Figures 5-7). The head part was then placed back in the incision and fixed with the radial neck region with two oblique screws of size 2 x 26 mm and 2 x 28 mm, thus resembling a tripod. Postoperatively, a slab was given which was extended above the elbow joint for three weeks, following which the patient was advised initiation of the range of motion. The final reduction was visualized under C-arm and was found to be satisfactory (Figures 8-10).

Follow-up and outcome

**Figure 11:**
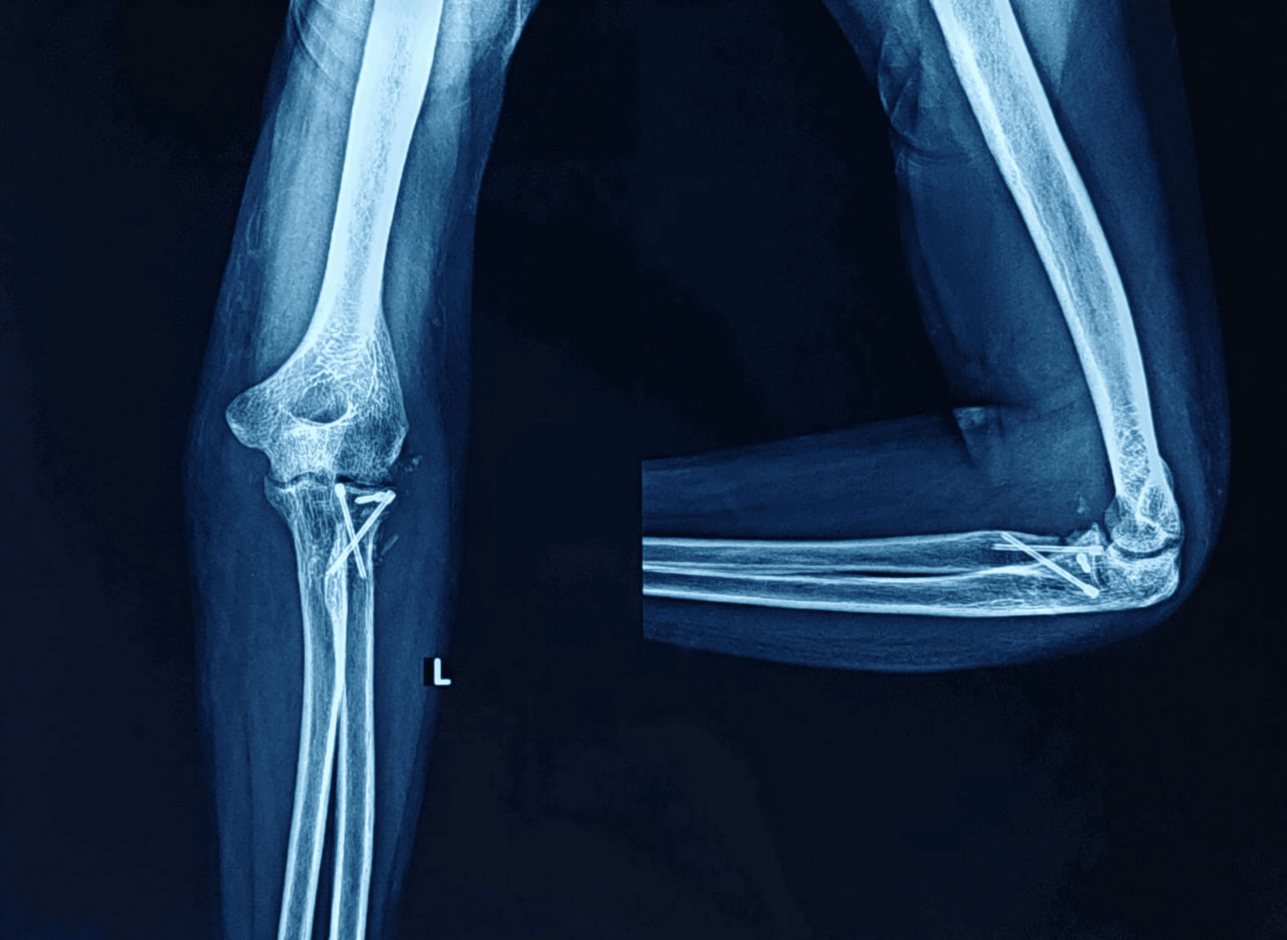
One-month follow-up X-ray depicting AP and lateral views of the left elbow joint AP: Anteroposterior

**Figure 12:**
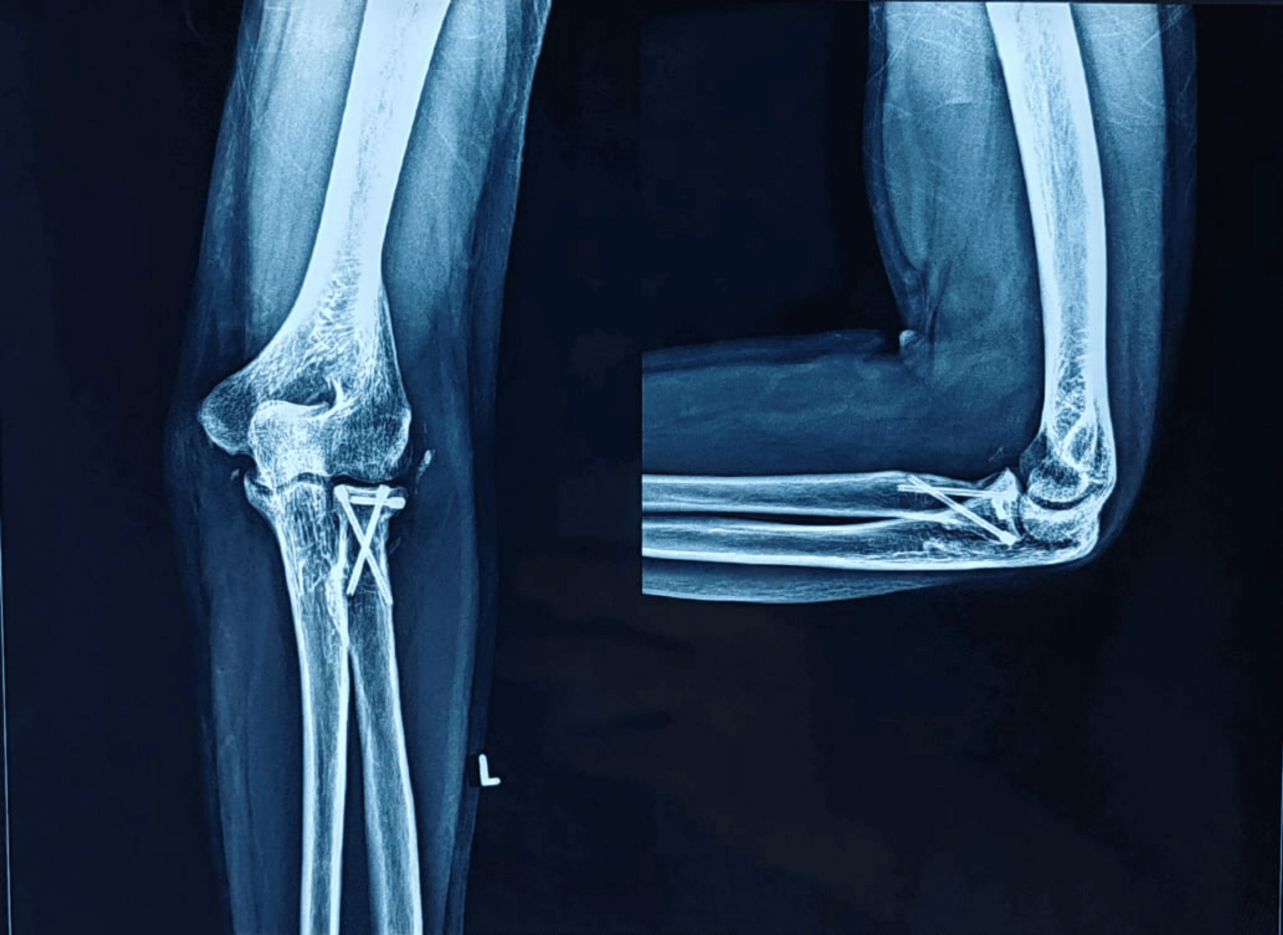
One-year follow-up X-ray depicting AP and lateral views of the left elbow joint AP: Anteroposterior

**Figure 13:**
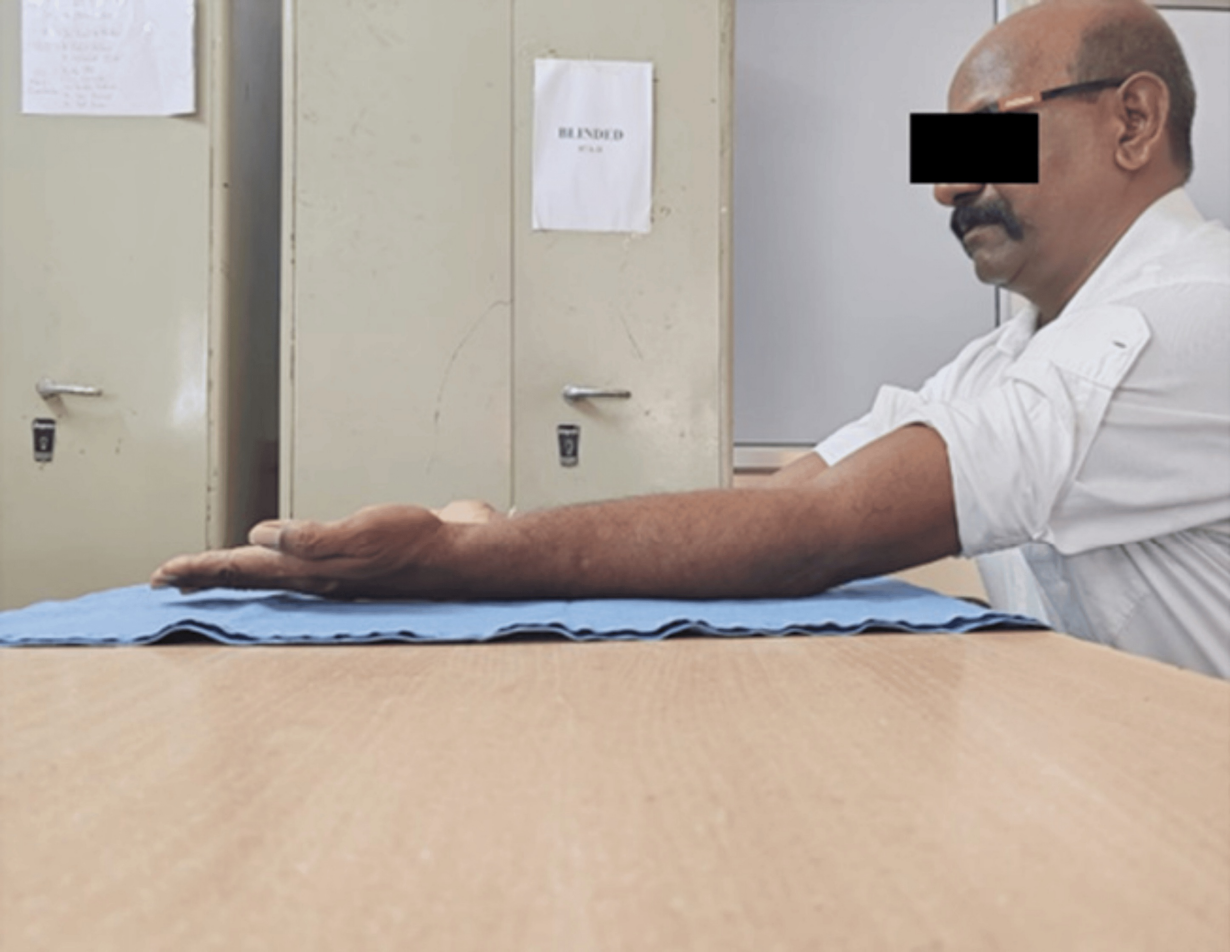
Picture showing postoperative range of motion of elbow extension after one year

**Figure 14:**
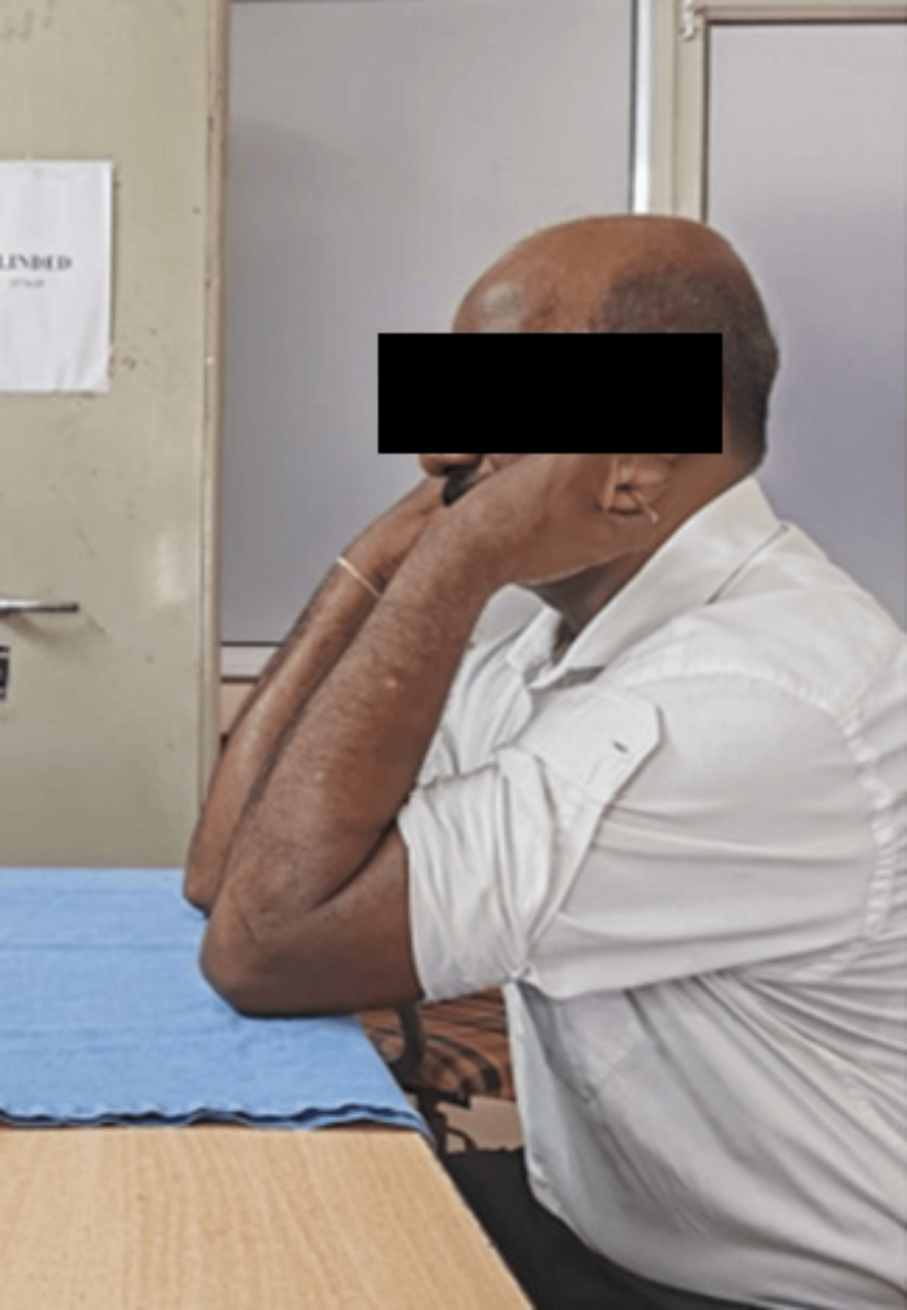
Picture showing postoperative range of motion of elbow flexion after one year

**Figure 15:**
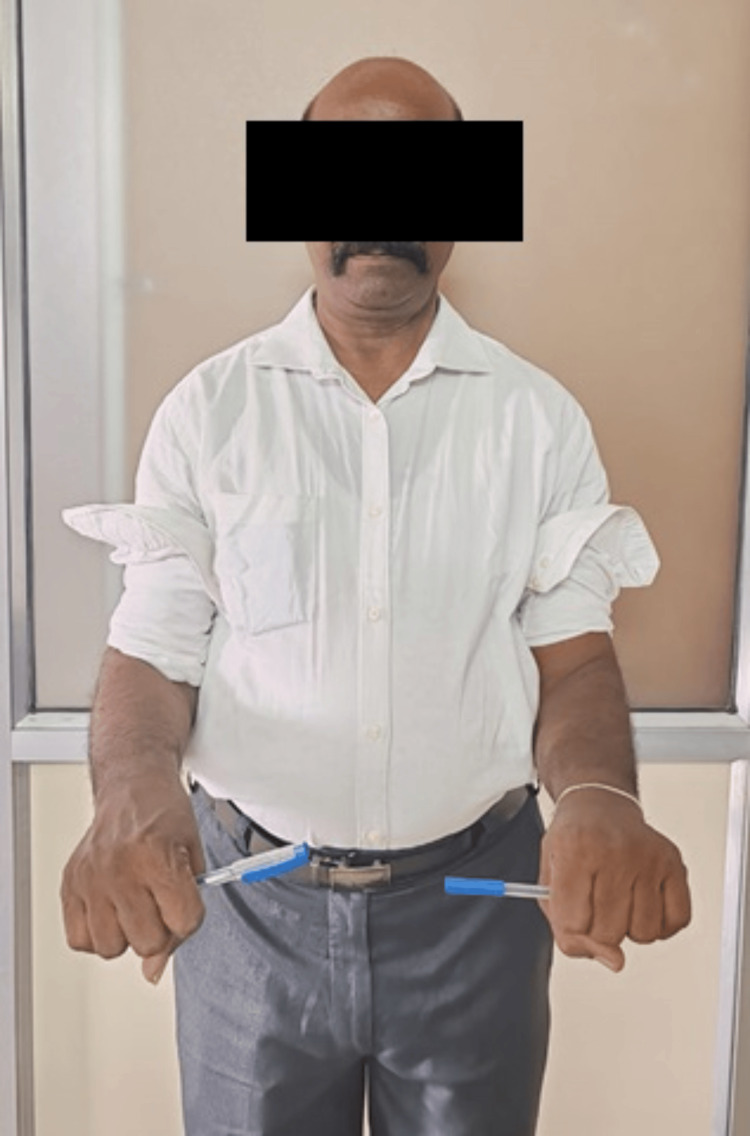
Picture depicting range of motion of forearm pronation after one year

**Figure 16:**
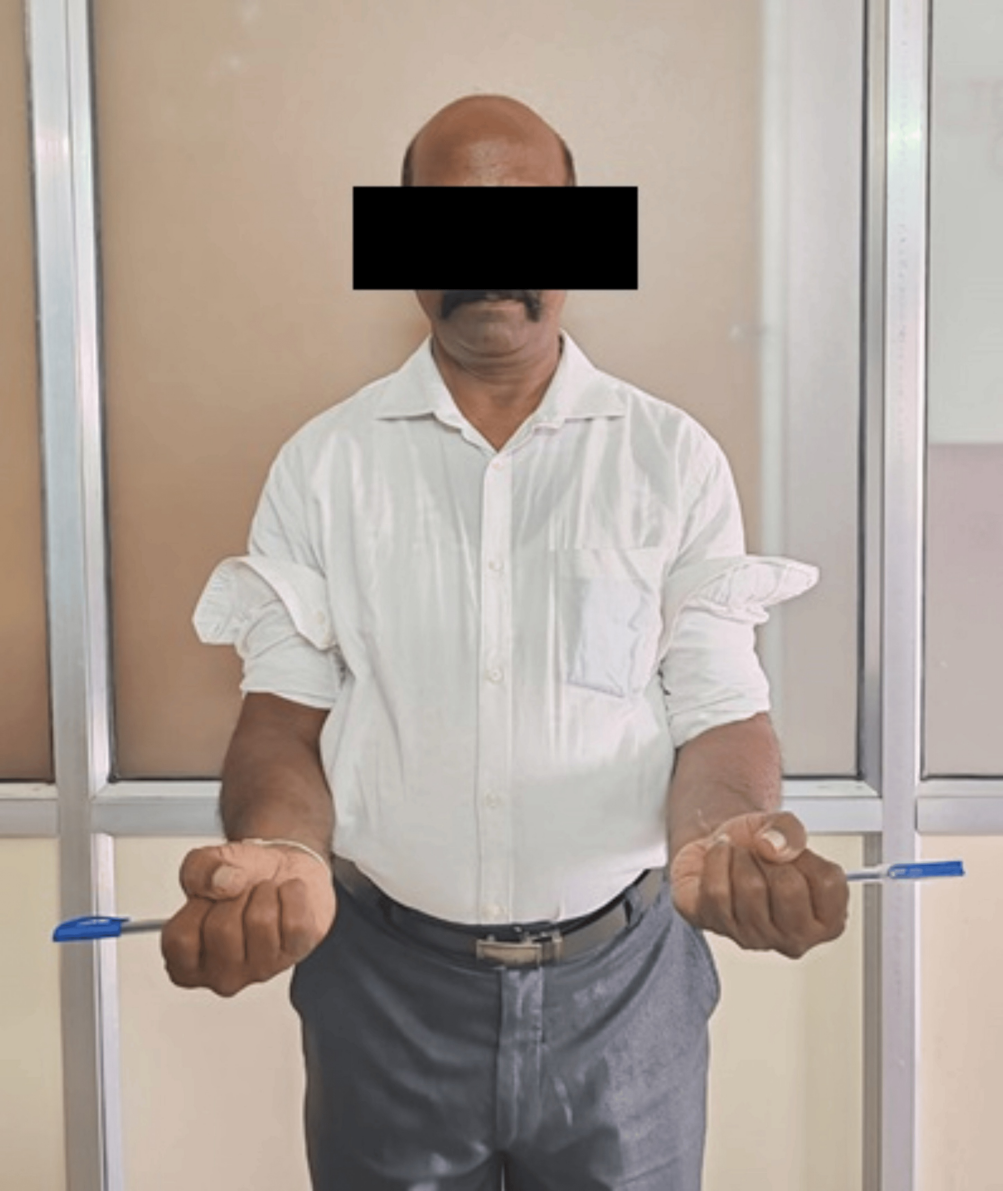
Picture depicting range of motion of forearm supination after one year

The slab was removed at the end of three weeks, and the patient was advised tolerable range of motion with gradual increments in the motion. Follow-ups were done regularly till one year. At follow-up, X-rays were obtained and the outcome determination was done on the basis of range of motion and Mayo Elbow score (Figures 11-12) [6]. The patient postoperatively had marked progress in the degree of motion at one-year follow-up. The subject had an elbow extension-flexion range of motion from 0° to 110° (Figures 13-14) (Video 1) and a complete degree of motion for supination and pronation (Figures 15-16). There was marked improvement in the elbow score seen with the follow-up with the score being fair (65) at one-month follow-up, following which the patient was advised physiotherapy and the scored improved to excellent (90) at six-month follow-up and 95 at one-year follow-up.

**Video 1:** One-year follow-up range of motion of the left elbow joint in a case of left radial head Mason type 4 fracture

An elbow performance score developed by Mayo clinic was used to determine the clinical outcome. It consists of various parameters such as intensity of pain, motion range, stability of the joint, and the function of the daily activities. The score can be given as excellent, good, fair, and poor [6].

## Discussion

Fractures of the head of the radius are quite common and are encountered due to elbow trauma and are usually accompanied by elbow dislocation. The head of the radius provides stability during valgus stress, transfers the axial load from the forearm to the arm, and is an important stabilizer when it acts along with the collateral ligaments [7]. The radial head is responsible for the transmission of 60% of the axial load from the radius to the humerus [6]. Management of the head of the radius is based on the rationale of its preservation [2].

Treatment modality depends grossly on the classification of the fractures and broadly consists of conservative management, internal fixation following open reduction which can be done with the use of compression screws which are headless or a plate, prostheses for the radial head, and its excision. While radial head arthroplasty is reserved for marked comminution [4], radial head excision is linked with its own set of complications and is doomed with disastrous results [7].

Wu et al., in 2016, found fixation of fractures using headless compression screws to provide good axial loading strength. They also attributed better results with screw fixation to the smaller surgical exposure and found the time to union to be shorter in these patients. They noted the Mayo elbow performance score to be 87.7 for the fixation with a headless compression screw and found it to be comparable with other treatment modalities [2].

Wu et al. found excellent results in the management of type 3 and 4 Mason fractures dealt with headless compression screws. The Mayo elbow score was higher for the treatment of type 3 fractures as compared with fractures which are type 4 (score of 89, i.e., good). A similar score of 95 was found in our present study. They started the advantage of management with headless compression screws over plates due to the ability to pass the screw beyond the safe margin to achieve maximal reduction [8].

A similar advantage was noticed by Lipman et al. in 2017 in their study, although they preferred the tripod technique for the management of Mason type 2 fractures [1]. Jordan et al., in 2017, noted the complexity of type 3 and 4 fractures with associated soft tissue injuries and stated the importance of management of these injuries simultaneously with the management of the fractures [3].

Model et al., in 2021, did a retrospective analysis, to study the tripod technique and its outcomes and found it to be a useful alternative. They found the results of fractures of the head of the radius with significant comminution, managed with plate following internal fixation after open reduction than internal fixation after open reduction with screw alone to be associated with a higher reporting of failure of hardware, nonunion, and a range of movement which was decreased [4]. They also noticed a lower union time and a lower rate of nonunion and heterotrophic ossification in patients managed with headless compression screws. Our current study presents similar results with good signs of union on the radiographs and improved range of motion on subsequent follow-ups. There was no sign of heterotrophic ossification clinically or radiologically at the follow-up.

Yano et al. stated the drawbacks after improper management of the fracture of the radial head such as the restriction in activities of the daily living pertaining to pain, restricted range of movement, and joint instability. They found similar results with plate and compression screws but found the prior procedure to be superior for comminuted radial head fractures. They recorded a Mayo elbow performance scoring of 95.9 (excellent) which they found to be similar to the plate group [6].

Radial head fracture management is associated with a steep learning curve and can be attributed to factors such as limited working space, vicinity to the posterior interosseous nerve, need for absolute reduction, and stability to avoid inappropriate reduction [8].

In our current scenario, the patient had a significant improvement in the range of movement in both the extension and flexion axis and the pronation and supination axis. The patient was able to get back to his livelihood with no obvious complications.

## Conclusions

Management of the fracture of the radial head seems controversial. A lot of surgeons have different management modalities depending on the presentation of the fracture, with the preference for radial head arthroplasty and radial head excision for Mason type 3b fractures. However, meticulous planning and careful dissection can help achieve a stable anatomical reduction using headless compression screws. The tripod technique can be used for high-grade fractures of the radial head, and a good functional outcome can be obtained.
